# Analysis of the effect of percutaneous vertebroplasty in the treatment of thoracolumbar Kümmell’s disease with or without bone cement leakage

**DOI:** 10.1186/s12891-020-03901-2

**Published:** 2021-01-05

**Authors:** Ya-Ping Xiao, Ming-Jian Bei, Cui-Qing Yan, Jian-Zhong Chang

**Affiliations:** 1 Department of Orthopedic Surgery, CR & WISCO General Hospital, Wuhan University of Science and Technology, No. 209 Yejin Road, Wuhan, 430000 Hubei Province People’s Republic of China; 2 Department of Orthopedic Surgery, Emergency General Hospital, Beijing, 100028 People’s Republic of China; 3 Department of Ultrasonography, Wuchang Hospital, Wuhan University of Science and Technology, Wuhan, 430000 People’s Republic of China

**Keywords:** Kümmell’s disease, Bone cement, Leakage, Vertebral compression fracture, Percutaneous vertebroplasty

## Abstract

**Background:**

Bone cement leakage is a major complication in the treatment of percutaneous vertebroplasty for Kümmell’s disease, and the focus of close attention during the surgery. The purpose of this article was to investigate the clinical outcomes of Kümmell’s disease treated by percutaneous vertebroplasty with or without bone cement leakage.

**Methods:**

A total of 64 patients with Kümmell’s disease from December 2016 to February 2018 treated by percutaneous vertebroplasty were included in the study. After the treatment, 32 cases were respectively divided into two groups according to X-ray examination of bone cement leakage: leakage group and non-leakage group. Preoperative course, age, sex, bone mineral density, damaged segment, anterior vertebral height, vertebral compression rate, Cobb angle, visual analogue scale and Oswestry dysfunction index were compared between the two groups. After surgery, the amount of bone cement injected, operation time, adjacent vertebral refracture rate, visual analogue scale, Oswestry dysfunction index, the recovery value of vertebral anterior height and the improvement value of Cobb angle were compared between the two groups.

**Results:**

The course, age and Cobb angle of the leakage group were significantly greater than those of the non-leakage group (*P*< 0.05, respectively). The height of anterior vertebral margin and bone mineral density in the leakage group were significantly lower than those in the non-leakage group (*P*< 0.05, respectively). The two groups were followed up for at least 24 months. The amount of bone cement injected was significantly greater in the leakage group than in the non-leakage group (*P*=0.000). Visual analogue scale and Oswestry dysfunction index of the two groups on the second day after surgery and at the last follow-up were significantly lower than these before surgery (*P*< 0.05, respectively), but there was no significant difference between the two groups. In the leakage group, the recovery value of the anterior edge height of the injured vertebra and the improvement value of the Cobb angle on the second day after surgery and at the last follow-up were significantly improved compared with the non-leakage group (*P*< 0.05, respectively).

**Conclusion:**

Percutaneous vertebroplasty is an effective and minimally invasive treatment for Kümmell’s disease. The leakage group had longer course, older age, more serious kyphotic deformity, vertebral compression and osteoporosis, and higher amount of bone cement injected than these of the non-leakage group. However, there were not significant differences in the rate of adjacent vertebral refractures, visual analogue scale and Oswestry dysfunction index between the two groups. Therefore, the bone cement leakage does not affect the surgical effect.

## Background

Kümmell’s disease (KD) is a relatively rare and special type of osteoporotic vertebral compression fractures (OVCFs) [1]. KD is also called post-traumatic vertebral osteonecrosis, avascular necrosis after OVCFs, intravertebral vacuum, vertebral pseudarthrosis, OVCFs nonunion, delayed vertebral collapse [2]. The incidence rate of KD was reported as high as from 12.1 to 42.4% in the OVCFs [3, 4]. The main clinical features are kyphosis and intractable pain of lumbago and lower back [5]. Delayed vertebral collapse and characteristic change of intravertebral vacuum phenomenon (IVP) or intravertebral vacuum cleft (IVC) can be found by imaging examination [6]. The IVP is usually presented as intravertebral radiolucent shadows in typically band like or linear shape, which is usually accompanied by peripheral sclerosis [7]. The limited fluid filling can be found in the vertebral IVC area by MRI examination.

Spontaneous reduction of the fractured vertebral body in KD patients can be occurred in the posterior extension of spine [8, 9]. Excessive reduction tends to accelerate the process of vertebral ischemia and avascular necrosis, and leads to severe recollapse [10]. Therefore, percutaneous vertebroplasty (PVP) has clinical selection advantages over percutaneous kyphoplasty (PKP) in the treatment of KD [11]. PVP can stabilize the fractured vertebral body in a minimally invasive manner, relieve quickly lumbago and back pain, restore partial height of the vertebral body, and correct kyphosis [12, 13]. PVP reduces the risk of cuneate deformity and nerve injury in KD patients, which suggests early surgical treatment [14]. However, bone cement leakage is a common complication of PVP in the treatment of KD, and it is also the focus of the surgeon to pay close attention to during the operation. At present, it has not been reported whether the bone cement leakage has its different clinical characteristics and will produce different clinical effects.

Therefore, the authors used a retrospective study method to analyze the single thoracolumbar segment KD patients treated with PVP with or without bone cement leakage and admitted to our hospitals from December 2016 to February 2018. The preoperative and postoperative clinical data were analyzed to evaluate the differences of clinical outcomes, so as to provide some references for clinical practice.

## Methods

### Selection criteria

This study is a retrospective study. The two groups were followed up for at least 24 months. The inclusion criteria were as follows: (1) Patients with single thoracolumbar segment segment KD; (2) The presence of IVP was confirmed by magnetic resonance imaging (MRI) or computed tomography (CT) [15]. (3) The pain site of the physical examination was same with the segment of imaging examination. (4) T value (Bone mineral density, BMD) of double-energy X-ray examination was less than − 2.5; (5) Follow-up time was more than 2 years without re-injury. The exclusion criteria were as follows: (1) Intolerance to surgery such as coagulation dysfunction, severe cardiopulmonary dysfunction, etc. (2) Surgical contraindications such as local or systemic infection; (3) Symptoms of nerve root and spinal cord compression; (4) KD or New OVCFs were found in other vertebral bodies. (5) The adjacent vertebral bodies were significantly compressed and flattened.

### General information

**Fig. 1:**
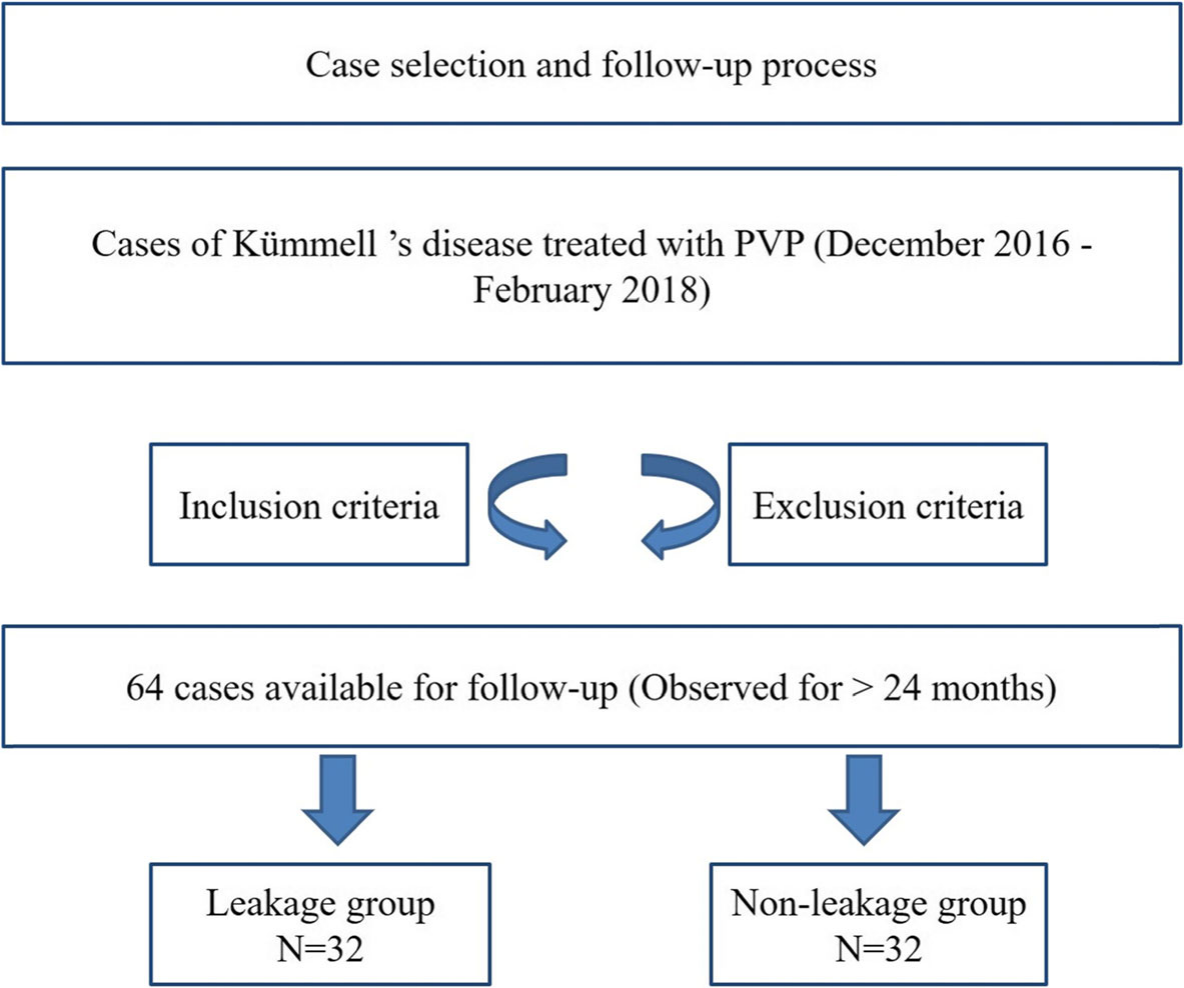
Case selection and follow-up process

A total of 64 patients with KD from December 2016 to February 2018 who met the selection criteria were selected in our study (Fig. 1). All of them received PVP treatment. There were 32 cases in the leakage group and 32 cases in the non-leakage group. The ethics committees of investigator’s hospitals approved this study. Oral informed consent of clinical data and images from all patients was obtained.

In our study, patients were primarily screened by X-ray imaging. For highly suspicious patients, MRI was used for diagnosis, and CT was used only for diagnosis of patients who could not use MRI examination.

We mainly observed whether there was leakage of bone cement after surgery through anteroposterior and lateral radiograph of the spine X-ray. For individual highly uncertain cases, CT examination of the spine may be used to determine the presence of bone cement leakage. If cement leakage is found, it is classified according to the anatomical location of the cement leakage.

### Surgical techniques

The procedure and the equipment used in this study were the same as in our previous studies. So please refer to our previous study for details of the procedure [11].

### Postoperative treatment

These patients remained in supine position for 24 h after surgery. In the first month after the surgery, these patients were mainly confined to bed rest, and could get out of bed intermittently with the protection of lumbar and back support. All patients were protected by lumbar and back support to get off the bed within 3 months after the surgery. Postoperative comprehensive antiosteoporosis treatment was as follows: oral administration of calcium carbonate D3 (600 mg /day), alendronate sodium tablets (10 mg/week) and intravenous infusion of zolefrononic acid (5 mg/ year). X-ray examination of injured vertebrae was performed on the 1st to 3rd day after the operation to understand the distribution of bone cement, and regular follow-up was conducted after discharge.

### Therapeutic evaluation

The amount of bone cement injected, operation time and the incidence of new adjacent vertebral fracture within 2 years were recorded. Visual analog scores (VAS) before surgery, on the second day after surgery, and at the last follow-up, were used to assess the level of pain in the lower back [16]. Oswestry dysfunction index (ODI) was used to assess the degree of activity function limitation [17]. The union rate and the rate of revision surgery would be recorded and evaluated during the follow-up.

The Cobb angle and anterior edge height of the injured vertebrae before operation, on the second day after operation and at the last follow-up were measured on the lateral X-ray film of the spine [18, 19]. The following indicators were calculated based on the above indicators: ① the recovery value of the anterior vertebral height = postoperative height of the anterior vertebra edge - preoperative height of the anterior vertebra edge; ② the improvement value of the Cobb angle = postoperative Cobb angle-preoperative Cobb angle.

### Statistical analysis

SPSS19.0 statistical software (IBM Corp., Armonk, NY, USA) was applied for statistical analysis. The measurement data were expressed as mean ± standard deviation. The Levene test was used for testing the homogeneity of variance. The independent sample T test was used for comparing the difference between the two groups. One-way ANOVA was used for comparing different time points between the two groups. Repeated measurement analysis of variance was used for comparing different time points within groups. Count data were used χ^2^ test for comparison between the two groups. *P* < 0.05 was considered statistically significant.

## Results

The gender, damaged segments, preoperative VAS and ODI between the two groups were not significant differences (all *P* > 0.05, Tables 1, 2). The course, age and Cobb angle of the leakage group were significantly greater than those of the non-leakage group (all *P*< 0.05, Table 1). The height of the anterior vertebral edge and BMD in the leakage group were significantly lower than those in the non-leakage group (*P* < 0.05, respectively, Table 1).

**Table 1:** Baseline data of the two groups

Parameters	Leakage group	Non-leakage group	*t*/χ^2^	*P*
Cases	32	32		
Gender			0.067	0.721
Male (cases)	9	8		
Female (cases)	24	23		
Age (years)	74.7±5.60	73.1±6.79	15.587	0.028
Course of disease (months)	3.5±1.44	3.2±1.35	7.864	0.001
Injured vertebral segment			1.372	0.751
T11(cases)	6	5		
T12(cases)	12	11		
L1(cases)	10	13		
L2(cases)	4	3		
BMD (T value)	−4.1±0.91	−3.9±0.69	2.428	0.038
Anterior edge height (mm)	11.00±4.19	11.7±3.80	−2.096	0.042
Cobb angle (°)	13.2±4.78	12.5±4.08	2.297	0.048

**Table 2:** Comparison of clinical outcomes between the two groups

Parameters	Leakage group	Non-leakage group	*t/*χ^2^	*P*
Follow-up time (months)	35.5±6.26	36.0±6.65	−0.087	0.734
Amount of bone cement injected (ml)	4.5±1.45	4.0±1.23	3.619	0.000
Operation time (min)	38.8±5.61	38.6±5.53	1.864	0.938
VAS scores				
Before surgery	8.0±1.41	7.8±1.42	2.696	0.362
On the second day after surgery	3.1±0.72*	3.2±0.76*	−1.494	0.719
At the last follow-up	3.2±0.78*	3.1±0.80*	1.031	0.726
ODI scores				
Before surgery	72.2±10.89	70.6±10.03	7.693	0.265
On the second day after surgery	25.4±5.42*	24.4±5.13*	3.537	0.654
At the last follow-up	26.9±5.41*	25.0±5.56*	12.872	0.126
Adjacent vertebral fractures(%)	18.75	15.63	0.110	0.740
Yes (cases)	6	5		
No (cases)	26	27		
The recovery value of anterior edge height (mm)				
On the second day after operation	3.4±1.70	2.1±1.28	2.572	0.000
At the last follow-up	3.1±1.56	2.0±3.47	1.956	0.000
The improvement value of Cobb angle (°)				
On the second day after operation	6.8±2.84	4.6±2.19	5.467	0.000
At the last follow-up	6.2±5.17	4.3±2.13	4.509	0.000

* Compared to before surgery, *P<* 0.05

Clinical symptoms of bone cement leakage were not reported in the leakage group. The leakage sites were as follows: 12 cases in the front of the vertebral body, 8 cases in the upper intervertebral space, 3 cases in the front of the vertebral body and upper intervertebral space, 5 cases in the lower intervertebral space, 2 cases in the front of the vertebral body and lower intervertebral space, 1 case in the front of the vertebral body and paravertebral vein, and 1 case in the paravertebral vein. There were no significant differences in the follow-up time, operation time, and the incidence of new adjacent vertebral fracture between the two groups (all *P*> 0.05). The amount of bone cement injected in the leakage group was significantly higher than that in the non-leakage group (*P*=0.000). VAS and ODI of the two groups were significantly lower on the second day after surgery and at the last follow-up than before surgery (*P*< 0.05), but there was not significantly difference between the two groups. Bone union was obtained in all cases both in the leakage group and non-leakage group at the last follow-up, and the union rate was 100%. None of the affected vertebrae in all cases both in the leakage group and non-leakage group was reoperated, and the rate of revision surgery was 0%. In the leakage group, the recovery value of the anterior edge height and the improvement value of Cobb angle of the injured vertebra were significantly improved compared with those of the non-leakage group (*P*< 0.05, respectively) (Table 2).

## Discussion

KD is a rare and special type of OVCFs. After mild trauma, vertebral ischemia and necrosis gradually lead to vertebral collapses, kyphosis and the formation of vertebral prosthesis. KD is usually manifested as intractable low back pain, and nerve injury may occur in severe cases [20, 21]. X-ray and CT examination can reveal the presence of IVC in the vertebral body, while MRI can show localized fluid filling in the vertebral IVC area [1, 2, 22]. IVC is mainly located in the thoracolumbar segment, and most fractures are wedge-shaped, occurring near the upper and lower endplate of the vertebrae [15, 23]. The IVC reduces the strength of the vertebral body and even is easy to cause recollapse. Kyphosis changes the physiological curvature of the spine, which is not conducive to the stability of the patient’s center of gravity. As a result, vertebral re-fractures are more likely to occur.

Due to the presence of pseudarthrosis in KD, the IVC and the cracks in the vertebral body can be widened during spinal stretch activities [24]. Partial correction of kyphosis and collapsed vertebral height can be obtained by posterior extension of the spine. The bone cement in the PVP surgery is usually confined to spread in the cracks, which has the effect of maintaining corrected height and kyphosis. Spontaneous reduction can be occurred in KD patients in the posterior extension of the spine without the use of balloon expansion of PKP surgery [8, 9]. Excessive reduction likely lead to accelerated process of vertebral ischemia and necrosis and even severe re-collapse [10]. Therefore, excessive reduction should be avoided during the operation to damage the vertebral body. PVP has been proven to be a safe and effective treatment for OVCFs. Patients can get out of bed early postoperatively and avoid the complications associated with long-term bedridden. Compared with open surgery, PVP has the advantages of minimally invasive and short operation time [25]. Chang et al. [11] have found that there is no significant difference in clinical efficacy between PKP and PVP in the treatment of KD, and PKP has no significant advantages. Considering the clinical cost, PVP has clinical superiority. Therefore, we used PVP surgery combined with postural reduction to treat KD patients.

Previous studies reported that there is not significant correlation between the amount of bone cement injected and pain relief, and it was even believed that 1.5 ml of bone cement injected into each vertebral body could obtain satisfactory pain relief [26, 27]. Biomechanical studies have confirmed that the strength of the vertebral body can be restored by the injection of about 2 ml bone cement for each vertebral body, and the stiffness of the vertebral body can be restored by the injection of about 4 ml bone cement for each vertebral body [27]. In this study, the amount of bone cement injected in both groups have reached the requirements to restore vertebral strength and stiffness. The amount of bone cement injected was significantly greater in the leakage group than in the non-leakage group, which may be related to the long course, serious degree of osteoporosis and vertebral compression, and longer formation of IVP in the leakage group. Therefore, for KD patients with long course and severe vertebral compression, intraoperative PVP treatment should be performed with caution, and the maximum injection volume and diffusion of bone cement should not be excessively pursued to reduce the leakage risk of bone cement.

In this study, none of the patients in the leakage group had neurological injury or other complications. VAS and ODI of the two groups were significantly lower after surgery than before the surgery, and maintained to the last follow-up. There was not statistical difference in improvement degree between the two groups, suggesting that there was not significant correlation between bone cement leakage and clinical efficacy. Similarly, after Li et al. [28] treated KD patients with bone cement treatment, VAS and ODI at the last follow-up were significantly improved compared with those before treatment. As for ODI score, the patient did not experience all life experiences of ODI questionnaire on the second day after surgery. When the patients answered the questionnaire on the second day after the surgery, they only inferred their relevant life experience of the ODI questionnaire based on their current pain level, which may be subject to subjective bias, so it should be treated with caution. According to the classification of the leakage site, the leakage site was mainly concentrated in the front of the vertebral body, followed by the intervertebral space and the paravertebral vein, rarely causing intraspinal leakage. Surgeons should carefully analyze the imaging data before surgery, fully understand the crack position and size, and grasp the location of pedicle injection to prevent the occurrence of bone cement leakage. Nieuwenhuijse et al. [29] found that the IVP of KD could extend beyond the vertebral body wall, and the injected bone cement could leak outside the vertebral body along this crack, increasing the risk of bone cement leakage. Therefore, it is necessary to closely observe the imaging data of injured vertebrae before operation to predict the location of bone cement leakage. Sclerosis bands are often formed around the IVP, which increases the difficulty of puncture. When violently breaking through the hardening band, the puncture needle may enter the front of the vertebral body, causing leakage risk to the front. It is necessary to determine the corresponding puncture angle and depth for different injured vertebrae, slowly inject bone cement in stages, understand the distribution of bone cement by intermittent fluoroscopy, and not blindly pursue the largest amount of bone cement injected.

The recovery value of the anterior edge height and the improvement value of Cobb angle of the injured vertebra in the leakage group were more obvious than those in the non-leakage group. Preoperative course, age, Cobb angle and vertebral compression rate of the leakage group were significantly higher than those of the non-leakage group, while the anterior vertebral height and BMD of the leakage group were significantly lower than those of the non-leakage group. These results suggested that the leakage group had more serious condition of KD than that of the non-leakage group, with large IVC volume and obvious pseudarthrosis formation. So, the crack space was large in the leakage group and the reposition of collapsed vertebral body was more obvious. After the cracks in vertebral body were filled with bone cement, bone cement injected can effectively restore the height of vertebral body and correct kyphosis.

The leakage group had an adjacent vertebral fractures of 18.75% vs. the non-leakage group 15.63%, but there was not statistical difference between the two groups. The rate of adjacent vertebral refractures in this study is roughly equivalent to previous studies. Yang et al. [30] reported that a range from 14.1 to 39.1% of OVCFs patients during the first year after PVP surgery experienced an adjacent vertebral fractures.

The limitations of this study are as follows. The first is the natural limitation of the retrospective study itself. There may be selection bias in the selection of cases. There may be recall bias and subjective bias in patients’ recall process. KD disease is relatively few, and it is easy to be complicated with adjacent vertebral fractures and multiple fractures. So, there are few cases to meet the selection criteria. Therefore, the small sample size in this study is needed to expand the sample size for further sample analysis. Due to equipment limitations, the three-dimensional measurement of IVC morphology and size was not performed before surgery, so there was a lack of further demonstration of the results in this study. The data collection researchers did not completely blind, and the data may be biased. Therefore, the current research outcomes provide a clinical reference, but further multi-center, randomized, double-blind clinical trials were needed to validate these findings.

## Conclusion

PVP can effectively relieve the pain, improve the function, effectively restore the vertebral height and correct kyphosis in the treatment of KD. Compared with the non-leakage group, the leakage group had longer course, older age, more serious kyphotic deformity, vertebral compression and osteoporosis, and higher amount of bone cement injected. However, there were no significant differences in the rates of adjacent vertebral refracture, VAS and ODI between the two groups. Therefore, the bone cement leakage does not affect the surgical effect.

## Data Availability

The datasets used and/or analysed during the current study are available from the corresponding author on reasonable request.

## Abbreviations

KD: Kümmell’s disease
PKP: Percutaneous kyphoplasty
PVP: Percutaneous vertebroplasty
OVCFs: Osteoporotic vertebral compression fractures
ODI: Oswestry disability index
IVC: Intravertebral vacuum cleft
BMD: Bone mineral density
VAS: Visual analogue scale
IVP: Intravertebral vacuum phenomenon
